# A two-sample bidirectional Mendelian randomization analysis investigates associations between gut microbiota and type 2 diabetes mellitus

**DOI:** 10.3389/fendo.2024.1313651

**Published:** 2024-03-01

**Authors:** Siyuan Song, Qiling Zhang, Li Zhang, Xiqiao Zhou, Jiangyi Yu

**Affiliations:** Department of Endocrinology, Jiangsu Province Hospital of Chinese Medicine, Affiliated Hospital of Nanjing University of Chinese Medicine, Nanjing, China

**Keywords:** gut microbiota, type 2 diabetes mellitus, *genus Lachnoclostridium Actinomyces*, bidirectional, metabolites

## Abstract

**Objective:**

This study sought to elucidate the causal association between gut microbiota (GM) composition and type 2 diabetes mellitus (T2DM) through a comprehensive two-sample bidirectional Mendelian randomization analysis.

**Method:**

T2DM data were sourced from the IEU OpenGWAS Project database, complemented by 211 gut microbiota (GM) datasets from the MiBioGen Federation. The primary analytical approach employed was inverse variance weighted (IVW), supplemented by MR-Egger regression and weighted median (WME) methods to investigate their potential interplay. Results were assessed using odds ratios (*OR*) and 95% confidence intervals (*CI*). The robustness and reliability of the findings were confirmed through leave-one-out analysis, heterogeneity testing, and assessment of horizontal pleiotropy. Furthermore, we explored the potential mediating role of metabolites in the pathway linking GM to T2DM.

**Result:**

A set of 11 Single Nucleotide Polymorphisms (SNPs) linked to GM were identified as instrumental variables (IVs). The IVW analysis revealed that increased abundance of the *genus Actinomyces, genus Bilophila, genus Lachnoclostridium, genus Ruminococcus gnavus group*, and *genus Streptococcus* corresponded to a heightened risk of T2DM. Conversely, higher levels of *genus Eubacterium oxidoreducens group, genus Oscillospira, genus Ruminococcaceae UCG003, genus Ruminococcaceae UCG010*, and *genus Sellimonas* were associated with a reduced risk of T2DM. However, following false discovery rate (FDR) correction, only the abundance of genus Lachnoclostridium retained a significant positive correlation with T2DM risk (*OR* = 1.22, *q value* = 0.09), while the other ten GM showed suggestive associations with T2DM. Reverse MR analysis did not reveal any causal relationship between T2DM and the increased risk associated with the identified GM. Additionally, metabolites did not exhibit mediating effects in this context.

**Conclusion:**

This study effectively pinpointed specific GM associated with T2DM, potentially paving the way for novel biomarkers in the prevention and treatment of this condition. The findings suggested that probiotics could emerge as a promising avenue for managing T2DM in the future. Furthermore, the analysis indicated that metabolites do not appear to act as mediators in the pathway from GM to T2DM.

## 1 Introduction

Type 2 diabetes mellitus (T2DM) is a metabolic disorder characterized by chronic hyperglycemia, primarily stemming from insulin resistance in peripheral tissues or inadequate insulin secretion from pancreatic islet *β* cells. Globally, the diabetic population has surged to approximately 420 million, with China alone exceeding 100 million individuals affected (1). Research findings indicate substantial alterations in the gut microbiota (GM) structure among T2DM patients compared to healthy individuals (2). GM actively participates in material and energy metabolism processes, influencing the onset and progression of metabolic disorders such as obesity through inflammatory responses, endotoxemia, and the production of short-chain fatty acids (SCFAs) (3). Research has demonstrated that individuals with T2DM exhibit an altered GM composition compared to healthy counterparts, characterized by an increase in *Firmicutes* abundance and a decrease in *Bacteroides* abundance. Moreover, the ratio of *Bacteroides to Firmicutes* has been positively correlated with plasma glucose levels measured by oral glucose tolerance test (OGTT) (4). Hwang (5) observed an elevated *Firmicutes to Bacteroides ratio* in the GM of diet-induced obese mice, which was associated with increased calorie absorption. This imbalance was implicated in perturbed glucose and lipid metabolism, altered gene expression, low-grade inflammation, and the pathogenesis of T2DM. Gou (6) developed a microbiome risk score (MRS) based on T2DM-associated gut microorganisms. They observed a positive correlation between MRS and glucose increment, providing evidence that dysbiosis of the GM contributes to aberrant glucose and lipid metabolism, thereby influencing the onset and progression of T2DM. The mycobiome and virome are recognized to exert significant, sometimes debilitating effects on T2DM. These components interact within the intestinal milieu. For instance, Bao (7) demonstrated that mitigating Candida albicans colonization represents a viable strategy for ameliorating T2DM progression. Similarly, viruses coexist within the human gut, and their interplay has been implicated in T2DM pathogenesis. The functional loss of several viruses and the disruption of virus-bacteria correlations suggest a potential role for enteroviruses in T2DM (8). Additionally, Gu (9) observed that Lactobacillus paracasei administration alleviated T2DM by modulating the intestinal microbiota SCFAs-hormone/inflammation axis. However, observational studies are susceptible to unknown confounders and reverse causality, thereby obscuring the causal relationship between GM and T2DM risk.

**Figure 1:**
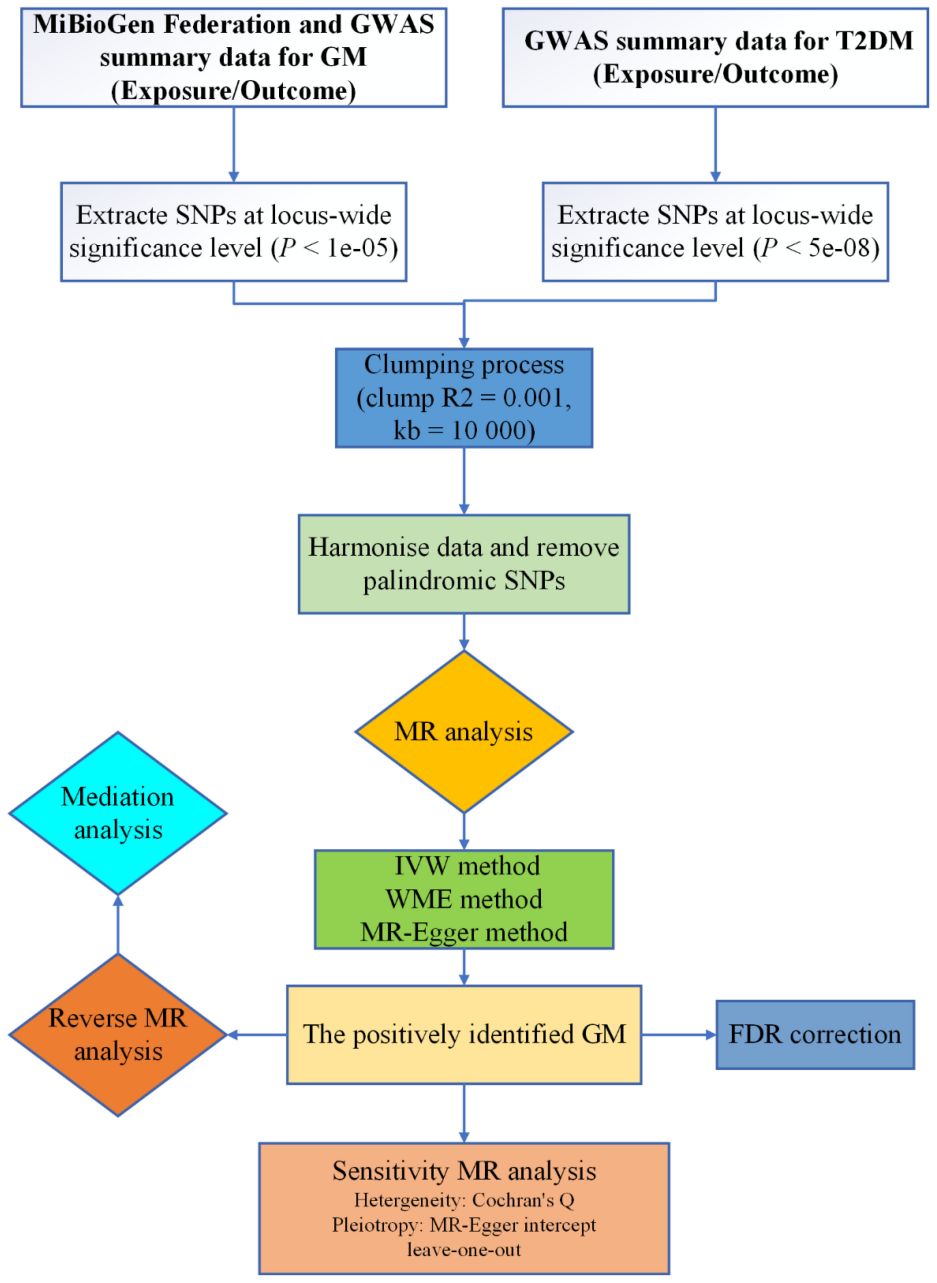
The protocol of our study procedure.

Mendelian randomization (MR) stands as an effective approach for discerning causal relationships between exposures and outcomes, leveraging single nucleotide polymorphisms (SNPs) as IVs (10). The inherent independence of MR ensures that genetic variations are generally uninfluenced by external factors or confounding variables, rendering the associations gleaned from MR analyses more robust than those from randomized clinical trials (RCTs) (11). In this investigation, data were gathered via genome-wide association studies (GWAS), and a two-sample bidirectional MR analysis was employed to elucidate the potential causal link between GM and the risk of T2DM, thereby furnishing genetic substantiation for their association. The schematic representation of our study protocol is depicted in **Figure 1**.

## 2 Materials and method

### 2.1 Study design

**Figure 2:**
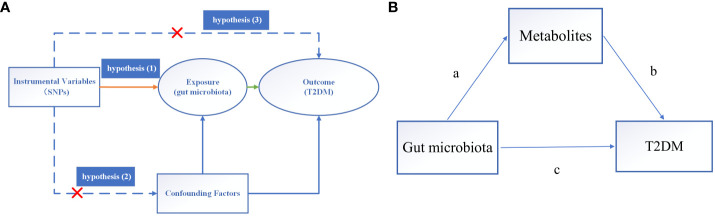
Overview of MR analysis. **(A)** The hub hypothesis of MR analysis. **(B)** Mediated analysis of metabolites from GM to T2DM pathway. (a) stands for the causal effect of GM on metabolites, and (b) stands for the causal effect of metabolites on T2DM, (c) represents the total influence of GM on T2DM.

In this investigation, GM was posited as the exposure variable, with SNPs exhibiting significant correlations selected as IVs, while T2DM was delineated as the outcome variable. Causal analysis was undertaken employing a two-sample MR analysis. The fulfillment of three hub hypotheses is imperative for robust MR analysis (12): (1) a substantial association between IVs and the exposure variable exists; (2) no confounding factors unduly influence the relationship between the exposure variable and the outcome variable; (3) IVs exert no direct effect on the outcome variable but instead operate solely through the exposure variable. These pivotal hypotheses are depicted in **Figure 2A**.

### 2.2 Data of exposure

The exposure data utilized in this study originated from a large-scale, multi-ethnic genome-wide meta-analysis conducted by the MiBioGen Federation. The MiBioGen Consortium aggregated 16s RNA gene sequencing maps and genotyping data from 18,340 subjects across 11 countries in Asia and Europe. Through meticulous analysis, characteristic loci associated with microbial groups were identified to ascertain genetic factors influencing the relative abundance or presence of microbial taxa, all of which were integrated into the genome-wide association study (GWAS) dataset. Within this GWAS endeavor, 211 subgroups of GM, categorized at the phylum level, were meticulously examined. Consequently, the genetic variation linked with 9 phyla, 16 classes, 20 orders, 35 families, and 131 genera of GM was delineated (13).

To delve deeper into the realm of GM sub-species, a query for “gut microbio” was executed within the IEU OpenGWAS Project, yielding 418 additional GM datasets. Subsequently, a secondary MR analysis was undertaken to corroborate the influence of GM sub-species on the risk of T2DM.

### 2.3 Data of outcome

The outcome data utilized in this study, designated as ebi-a-GCST006867, was procured from the IEU OpenGWAS Project website (gwas.mrcieu.ac.uk). The T2DM dataset encompasses a substantial sample size of 655,666 individuals, with a corresponding SNP count of 5,030,727. As all data utilized in this research are sourced from publicly available databases, no additional ethical approval was deemed necessary.

### 2.4 Tool variable filtering

We conducted a rigorous selection process for SNPs with significant correlation, employing a threshold of *P* < 1e-05 (14). To mitigate the influence of linkage disequilibrium (LD), we set parameters of *R^2^* = 0.001 and kb = 10,000. Additionally, all palindrome SNPs were excluded to ensure data integrity (15). To fulfill the second MR hypothesis, known as the independence hypothesis, we utilized the PhenoScanner (http://www.phenoscanner.medschl.cam.ac.uk/) database (16) to identify all eligible SNPs. SNPs associated with confounding factors related to T2DM, such as fasting insulin, HbA1c, and two-hour glucose challenge, were meticulously excluded from the analysis. The strength of the correlation between loci and exposure variables was assessed based on the *F* value of each SNP, with an *F* value exceeding 10 generally considered indicative of unbiased IVs (17).

### 2.5 Statistical analysis

#### 2.5.1 MR analysis

We employed multiple methods including the IVW method, WME method, MR-Egger regression, and forest plot analysis to thoroughly investigate the potential relationship between GM and T2DM, ensuring robustness of the findings (18). The IVW method, assuming all SNPs are valid IVs, offers the most precise effect estimation (10). MR-Egger regression, while capable of detecting and adjusting for pleiotropy, often yields less accurate estimates (19). The WME method, predicated on the assumption that at least 50% of IVs are valid, provides accurate estimation results (20). Given the superior efficiency of the IVW method compared to other MR methods, it was selected as the primary approach to assess causal effects in this study (21). A significance level of *P* < 0.05 was considered indicative of a causal relationship between exposure (GM) and outcome (T2DM) (22). To mitigate the risk of one kind of error and ascertain whether the findings were influenced by multiple testing, we also employed the *q value* to correct the false discovery rate (FDR) of GM. A *q value* of less than 0.1 indicates a positive correlation (23). When *P* < 0.05 but *q* ≥ 0.1, GM and T2DM are deemed suggestive of association.

#### 2.5.2 Sensitivity analysis

Cochran’s Q statistic was employed to assess heterogeneity. A *P*-value greater than or equal to 0.05 suggests no heterogeneity in causal analysis (19). Additionally, the funnel plot was utilized to detect heterogeneity, with symmetrical distribution of SNPs indicating absence of heterogeneity in the results (24). MR-Egger intercept analysis was utilized to assess pleiotropy between IVs and other potential confounding factors, ensuring that the selected IVs do not exert effects on outcome variables via pathways other than exposure factors. Statistical significance (*P* < 0.05) in MR-Egger intercept analysis indicates presence of horizontal pleiotropy (25). Furthermore, to evaluate the impact of each SNP, a leave-one-out (LOO) analysis was conducted to ascertain the comprehensive impact of individual SNPs (26). Results were reported as odds ratios (*OR*) with corresponding 95% confidence intervals (*CI*), with statistical significance set at *P* < 0.05.

#### 2.5.3 Bidirectional MR analysis

We conducted a two-sample bidirectional MR analysis to explore the reverse causal relationship between T2DM (exposure) and positively identified GM (outcome). The steps of bidirectional MR analysis mirror those of the MR analysis.

#### 2.5.4 Mediation analysis

To explore whether the influence of GM on T2DM could be mediated through metabolites, we examined the mediation of metabolites in the pathway from GM to T2DM (**Figure 2B**). We acquired GWAS data for 575 metabolites from the IEU OpenGWAS Project (with criteria of *P* < 5e-06, *R^2^ = *0.001, and kb = 10,000).

#### 2.5.5 Statistical software

All MR analyses were conducted using R (version 4.3.1) and the TwoSampleMR package.

## 3 Results

### 3.1 Tool variable filtering

**Figure 3:**
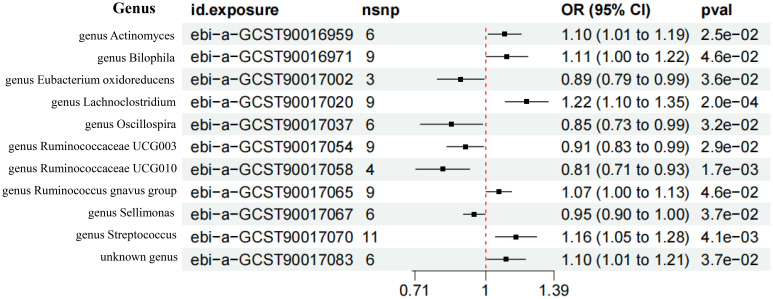
Forest plot of the MR results of 11 GM. nsnp represents the number of single nucleotide polymorphism. pval represents P-value.

According to the screening criteria of IVs, 6, 9, 3, 9, 6, 9, 4, 9, 6, 11, and 6 SNPs were extracted from the *genus Actinomyces, genus Bilophila, genus Eubacterium oxidoreducens group, genus Lachnoclostridium, genus Oscillospira, genus Ruminococcaceae UCG003, genus Ruminococcaceae UCG010, genus Ruminococcus gnavus group, genus Sellimonas, genus Streptococcus*, and *unknown genus*. The *F* statistics of the IVs included were all greater than 10, indicating that the bias of weak IVs would not have a substantial impact on the results (**Supplementary Table 1**; **Figure 3**).

### 3.2 MR analysis

**Figure 4:**
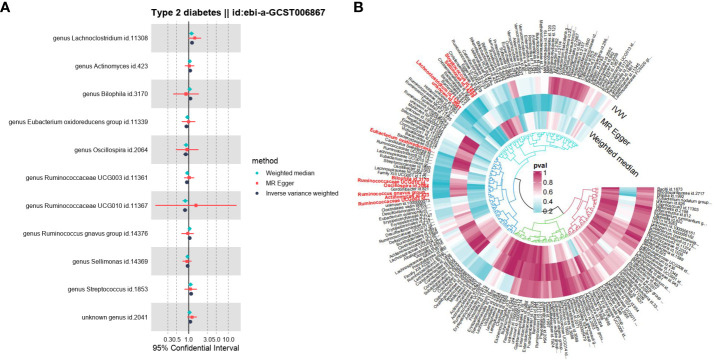
MR estimates of the causal effects of 11 GM on T2DM. Forest plot of estimates of the causal effects of 11 GM on T2DM. **(A)** The green triangle represents the WME method, the red square represents the MR-Egger method, and the grey circle represents the IVW method. **(B)** Circular graph of estimates of the causal effects of 11 GM on T2DM.

**Figure 5:**
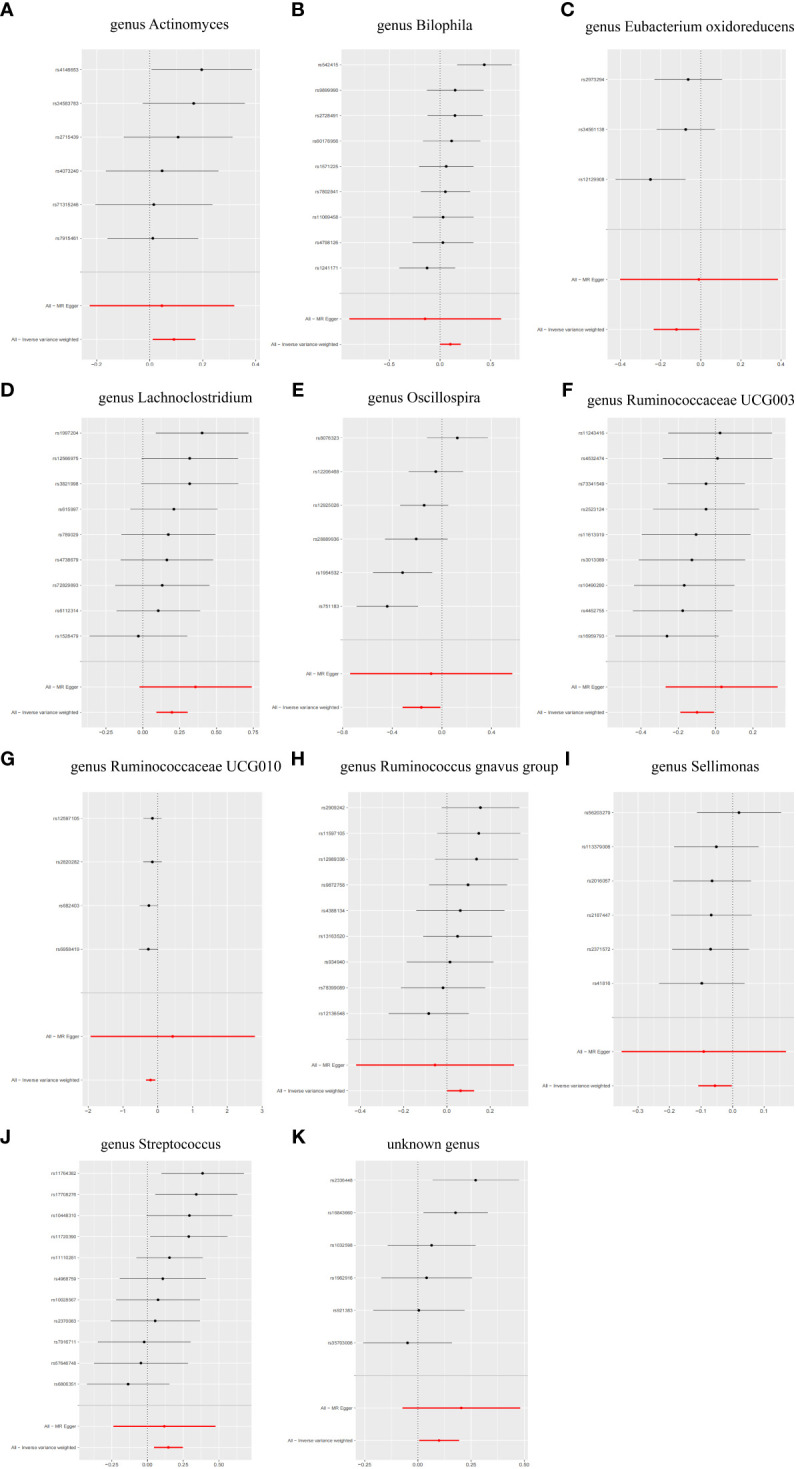
Forest plot of single SNP MR results. **(A)** genus Actinomyces. **(B)** genus Bilophila. **(C)** genus Eubacterium oxidoreducens. **(D)** genus Lachnoclostridium. **(E)** genus Oscillospira. **(F)** genus Ruminococcaceae UCG003. **(G)** genus Ruminococcaceae UCG010. **(H)** genus Ruminococcus gnavus group. **(I)** genus Sellimonas. **(J)** genus Streptococcus. **(K)** unknown genus.

**Figure 6:**
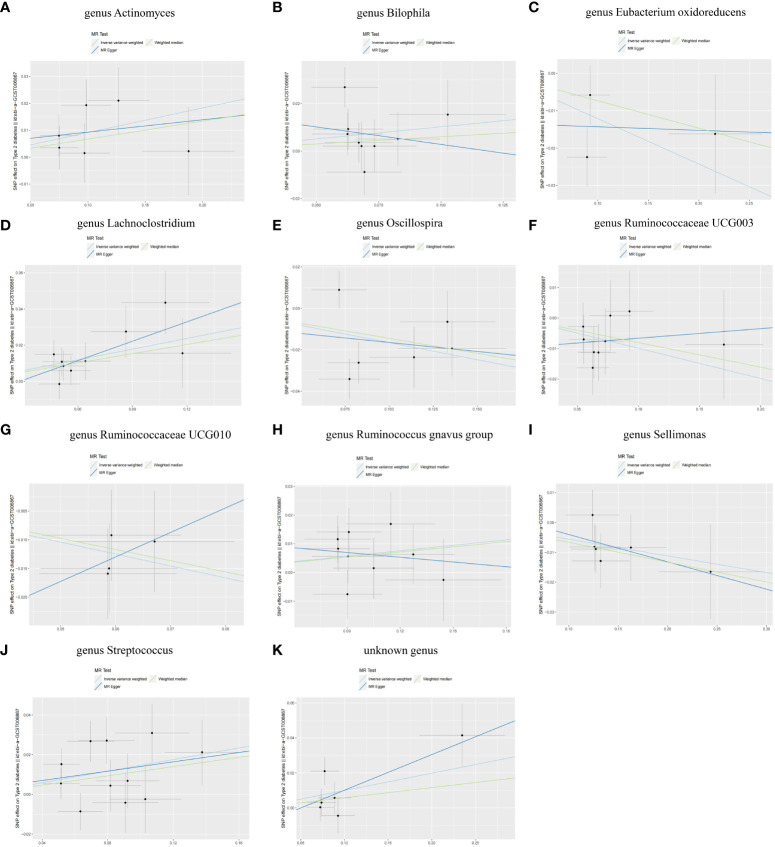
Scatter plots of SNP analysis **(A)** genus Actinomyces. **(B)** genus Bilophila. **(C)** genus Eubacterium oxidoreducens. **(D)** genus Lachnoclostridium. **(E)** genus Oscillospira. **(F)** genus Ruminococcaceae UCG003. **(G)** genus Ruminococcaceae UCG010. **(H)** genus Ruminococcus gnavus group. **(I)** genus Sellimonas. **(J)** genus Streptococcus. **(K)** unknown genus. The X-axis represents the influence of SNP on GM, the Y-axis represents the influence of SNP on T2DM, the black dot represents a single SNP, the line segment represents 95% *CI*, and the slope of the straight line represents the causal estimation of the MR method. The light blue line represents IVW method, the blue line represents MR Egger method, and the green line represents WME method.

The IVW analysis showed that the *genus Actinomyces* (*OR* = 1.10, 95%*CI* 1.01-1.19, *P* = 2.5e-02), *genus Bilophila* (*OR* = 1.11, 95%*CI* 1.00-1.22, *P* = 4.6e-02), *genus Lachnoclostridium* (*OR* = 1.22, 95%*CI* 1.10-1.35, *P* = 2.0e-04), *genus Ruminococcus gnavus group* (*OR* = 1.07, 95%*CI* 1.00-1.13, *P* = 4.6e-02), *genus Streptococcus* (*OR* = 1.16, 95%*CI* 1.05-1.28, *P* = 4.1e-03), and *unknown genus* (*OR* = 1.10, 95%*CI* 1.01-1.21, *P* = 3.7e-02) had a causal relationship with the increased risk of T2DM, while the *genus Eubacterium oxidoreducens group* (*OR* = 0.89, 95%*CI* 0.79-0.99, *P* = 3.6e-02), *genus Oscillospira* (*OR* = 0.85, 95%*CI* 0.73-0.99, *P* = 3.2e-02), *genus Ruminococcaceae UCG003* (*OR* = 0.91, 95%*CI* 0.83-0.99, *P* = 2.9e-02), *genus Ruminococcaceae UCG010* (*OR* = 0.81, 95%*CI* 0.71-0.93, *P* = 1.7e-03), and *genus Sellimonas* (*OR* = 0.95, 95%*CI* 0.90-1.00, *P* = 3.7e-02) had a causal relationship with the reduced risk of T2DM (**Figures 4A, B**). The results of the WME analysis supported the above conclusions. In the results of the *genus Bilophila, genus Ruminococcaceae UCG003, genus Ruminococcaceae UCG010, genus Ruminococcus gnavus group*, the total effect values of MR-Egger and IVW are in the opposite direction. (**Supplementary Table 2**; **Figures 5**, **6**). Because the results of IVW analysis are the most valuable and consistent with the forest plot, it was considered that the 11 GM we identified are related to the onset of T2DM. However, after FDR correction, it was found that only the *genus Lachnoclostridium* had a positive correlation with the risk of T2DM (*OR* = 1.22, *q value* = 0.09). The other ten GM were considered to be suggestive of T2DM.

Following the second MR analysis involving 418 GM sub-species and T2DM, we identified several taxa with potential causal relationships. Specifically, the *genera Lachnoclostridium, Ruminococcustorquesgroup*, and *Streptococcus*, along with *Betaproteobacteria, Micrococcaceae, Bacteroidales, Rothia, Bacteroidales (specifically Roseburia), Burkholderiales*, and *Bacteroidales_bacterium_ph8* were associated with an increased risk of T2DM. Conversely, the *family Alcaligenaceae*, along with the *genera RuminococcaceaeUCG003* and *RuminococcaceaeUCG010, Clostridiaceae*, and *Coprobacter_fastidiosus* exhibited a causal relationship with a reduced risk of T2DM (**Supplementary Figure 1**).

### 3.3 Sensitivity analysis

Cochran’s Q test and MR-Egger regression results showed that there is no significant heterogeneity and pleiotropy in this study (*P* > 0.05) (**Supplementary Table 3**). Funnel plots showed that the possible interference factors are less likely to have an impact on causality (**Supplementary Figures 2A-K**). LOO analysis showed that the remaining SNPs had no significant effect on the analysis results after removing individual SNP in turn (**Supplementary Figures 3A-K**). These analyses proved the robustness of the results of this study.

### 3.4 Bidirectional MR analysis

**Figure 7:**
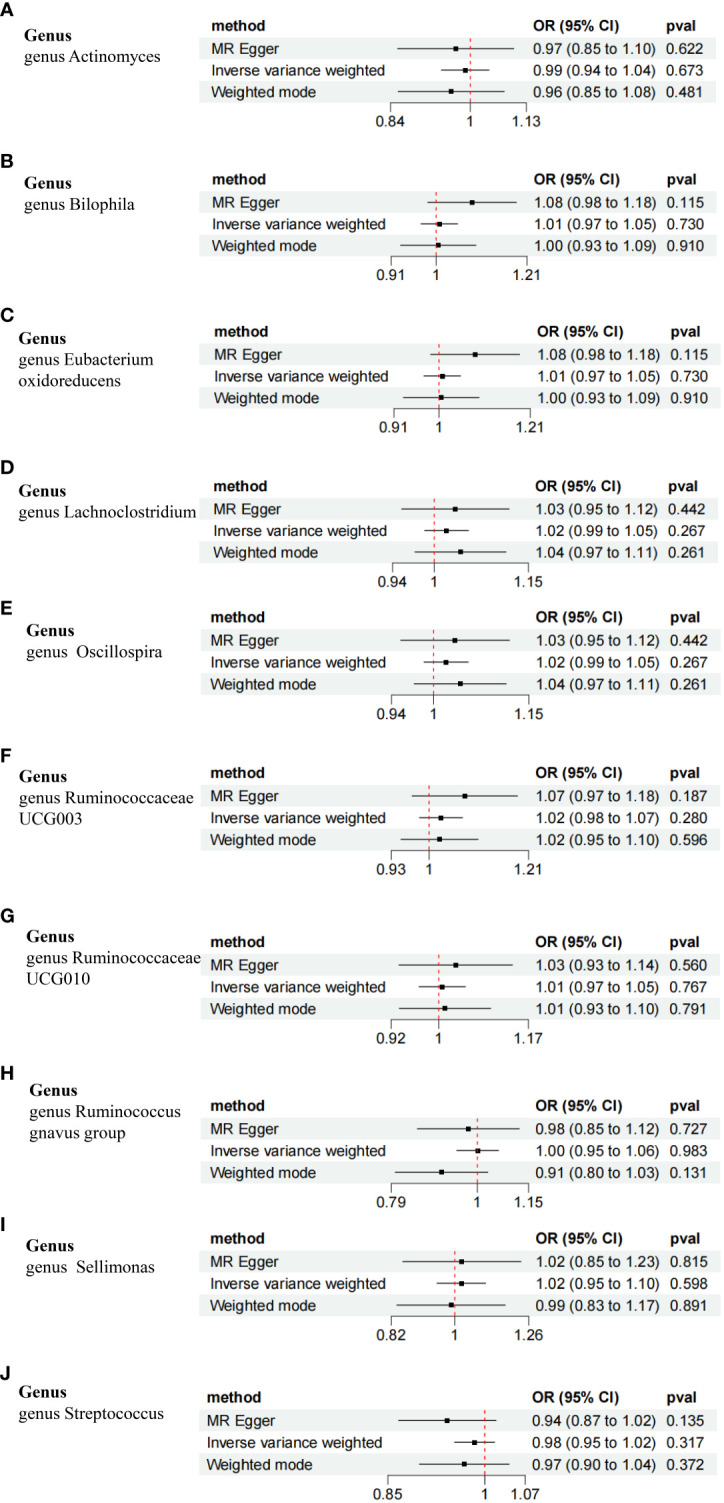
Forest plot of the bidirectional MR analysis of the positively identified 10 GM. **(A)** genus Actinomyces. **(B)** genus Bilophila. **(C)** genus Eubacterium oxidoreducens. **(D)** genus Lachnoclostridium. **(E)** genus Oscillospira. **(F)** genus Ruminococcaceae UCG003. **(G)** genus Ruminococcaceae UCG010. **(H)** genus Ruminococcus gnavus group. **(I)** genus Sellimonas. **(J)** genus Streptococcus.

The reverse MR analysis revealed no evidence of a causal relationship between T2DM and the heightened risk associated with the positively identified GM (**Figures 7A-J**). Notably, the unknown genus was excluded from this analysis.

### 3.5 Mediation analysis

**Figure 8:**
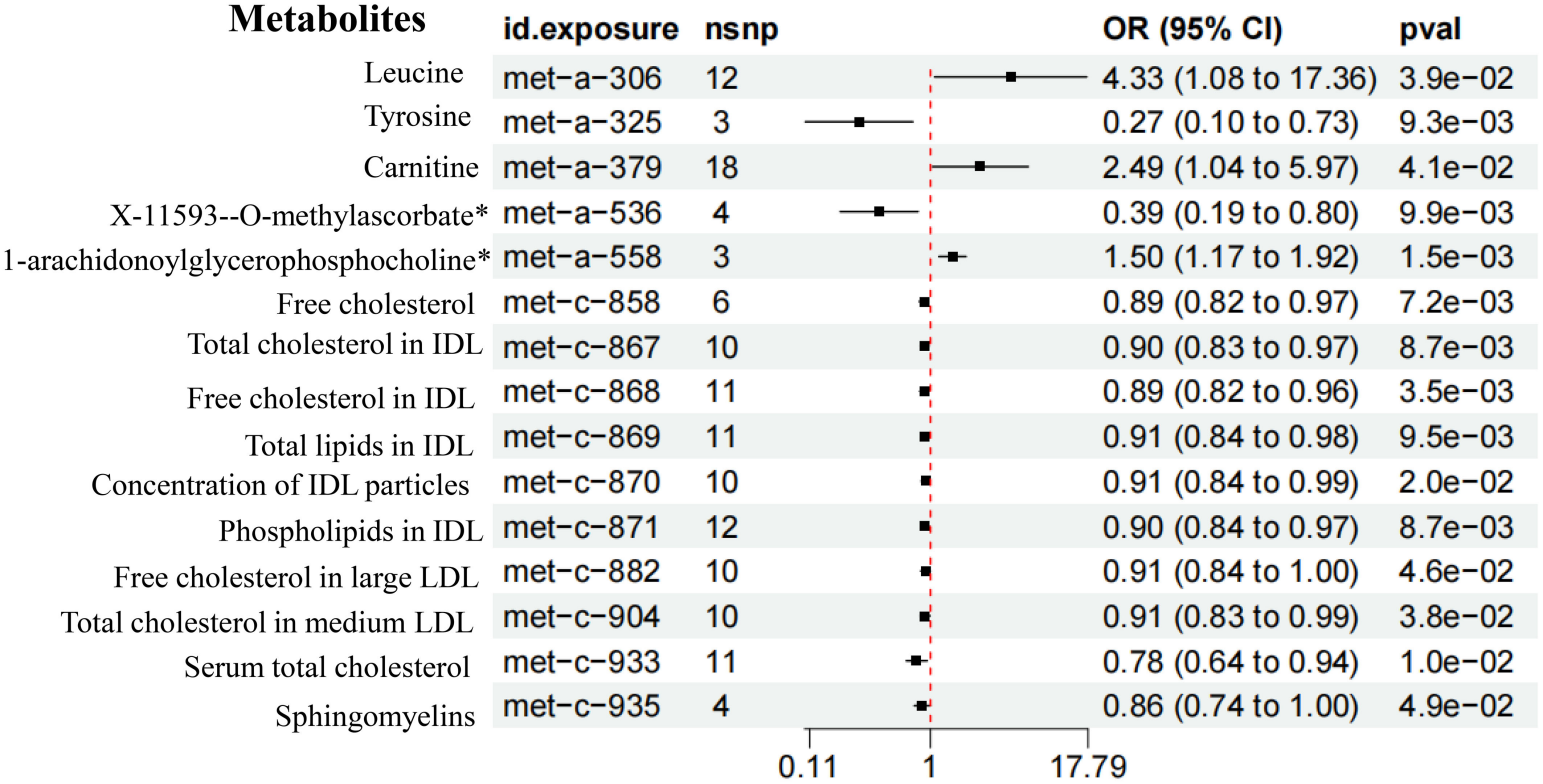
Forest plot of the MR results of metabolites on T2DM.

Through mediation analysis, we found that Leucine (*OR* = 4.33, 95%*CI* 1.08-17.36, *P* = 3.9e-02), Carnitine (*OR* = 2.49, 95%*CI* 1.04-5.97, *P* = 4.1e-02), and 1-arachidonoylglycerophosphocholine (*OR* = 1.50, 95%*CI* 1.17-1.92, *P* =1.5e-03) had a causal relationship with the increased risk of T2DM (**Figure 8**). Both GM and metabolites exhibit causal effects on T2DM. Although metabolites appear to mediate the relationship between GM and T2DM, the requirement for mediation is a significant association between GM and metabolites. However, our results indicate no causal relationship between GM associated with T2DM and metabolites related to T2DM. This suggested that metabolites do not act as mediators in the pathway from GM to T2DM (**Supplementary Table 4**).

## 4 Discussion

In this study, based on large-scale GWAS data, a two-sample MR method was used to analyze the causal relationship between GM and T2DM. The results showed that *genus Actinomyces, genus Bilophila, genus Lachnoclostridium, genus Ruminococcus gnavus group*, and *genus Streptococcus* had a causal relationship with the increased risk of T2DM, while *genus Eubacterium oxidoreducens group, genus Oscillospira, genus Ruminococcaceae UCG003, genus Ruminococcaceae UCG010*, and *genus Sellimonas* had a causal relationship with the reduced risk of T2DM. Further sensitivity analysis results indicated that the above results are consistent and reliable.

Research indicates a close relationship between T2DM and GM, with GM’s structural composition and metabolites exerting influence on T2DM (27). *Actinomycetes*, known for their efficacy in steroid conversion, play a pivotal role in this process (28). Specifically, cholesterol in the liver undergoes conversion into primary cholic acid, subsequently forming secondary cholic acid through enterohepatic circulation. Notably, secondary cholic acid, a predominant metabolite of intestinal bacteria, has been implicated in regulating glucose homeostasis via bile acid sensing mediated by Takeda G protein-coupled receptor 5 (TGR5) (29). Activation of TGR5 by bile acid prompts an increase in energy expenditure, thereby mitigating diet-induced obesity. Furthermore, TGR5 signaling stimulates the release of intestinal glucagon-like peptide-1 (GLP-1), enhancing liver and pancreas function and improving glucose tolerance in obese mice (30).

*Bilophila* plays a multifaceted role in gut health, contributing to both intestinal inflammation and disturbances in bile acid metabolism and microbial composition. These disruptions ultimately culminate in metabolic impairment and host dysfunction. Notably, *Bilophila* exhibits a negative correlation with glucose metabolism, indicating its involvement in regulating susceptibility not only to inflammatory diseases but also to metabolic disorders (31). These findings underscore the intricate interplay between GM and energy metabolism, highlighting their correlation with clinical indicators.

Blandino highlighted a positive correlation between the abundance of the *genus Lachnoclostridium* and fasting blood glucose as well as glycosylated hemoglobin levels (32). Elevated levels of *Lachnoclostridium* are associated with decreased circulating acetic acid, potentially leading to increased abdominal fat accumulation and exerting a detrimental effect on T2DM.

*Ruminococcaceae*-producing ursodeoxycholic acid (UDCA) plays a key role in promoting bile acid secretion. In a study investigating the impact of exercise on insulin sensitivity and glucose homeostasis in individuals with pre-T2DM, findings revealed a negative correlation between the abundance of *Ruminococcaceae* and fasting plasma glucose (FPG), fasting insulin levels, and homeostatic model assessment of insulin resistance (HOMA-IR). Conversely, a positive correlation was observed with the insulin sensitivity index (33). Moreover, enhanced jejunal-ileal bypass surgery in Goto-Kakizaki rats led to significant reductions in weight gain and serum lipid levels, accompanied by marked improvements in pancreatic islet *β*-cell function, glucose tolerance, and insulin resistance. Postoperatively, there was an increase in the abundance of *Ruminococcaceae*, which exhibited a negative correlation with blood glucose levels and a positive correlation with insulin levels (34).

*Streptococcus* is positively correlated with insulin, connexin, and the steady-state model evaluation index of insulin resistance (35). SCFAs represent crucial metabolites synthesized by beneficial microorganisms (36). They exert influence on glucose homeostasis by modulating glucose absorption and utilization across various organs (37). Research has indicated a correlation between alterations in SCFA levels in diabetic mice and the regulation of lipopolysaccharide-binding protein by *Streptococcus* (38). SCFAs have demonstrated favorable effects on T2DM by diminishing the production of pro-inflammatory cytokines such as TNK-α, IL-6, MCP-1, and NF-κB (39). Notably, this study marks the first instance of identifying the *Eubacterium oxidoreducens group, genus Ruminococcaceae UCG003*, and *genus Ruminococcaceae UCG010* as negative regulators in the progression of T2DM. Additionally, there is scant literature on the relationship between the *Eubacterium oxidoreducens group* and T2DM.

*Oscillospira* is negatively correlated with fasting blood glucose, which is an index of glucose metabolism disorder (40). In addition, *Oscillospira* can produce butyrate (41). Julienne Siptroth described the fermentation to butyrate for T2DM, which tend to have high potential for disease detection (42). *Oscillospira* plays an important role in GM, and its abundance is closely related to host health (43).

*Sellimonas*, identified as a Gram-positive obligate anaerobe (44), exhibits increased relative abundance in individuals recovering from intestinal ecological disturbances triggered by tumors or chronic metabolic ailments, eventually stabilizing at a steady state (45, 46). Consequently, *Sellimonas* emerges as a potential biomarker candidate for the restoration of intestinal homeostasis.

In this study, the relative abundance expression of GM was employed to delineate their potential “beneficial” or “harmful” roles in the context of T2DM. However, the precise mechanisms underlying how GM contribute to the development of T2DM remain elusive. It was postulated that metabolites could serve as the mediating factor between GM and T2DM. Our findings identified Leucine, Carnitine, and 1-arachidonoylglycerophosphocholine as causally linked to an increased risk of T2DM. Nonetheless, our analysis revealed that metabolites did not function as mediators in the pathway from GM to T2DM.

Recent studies have delved into the impact of GM on the etiology of T2DM. The SCFA theory, bile acid theory, and endotoxin theory represent three potential mechanisms through which GM may contribute to the onset and progression of T2DM. Based on the findings of this investigation, we postulate that the *genus Actinomyces, genus Bilophila, genus Lachnoclostridium, genus Ruminococcus gnavus group*, and *genus Streptococcus* may disrupt pancreatic islet cell function by influencing the metabolism of SCFAs, bile acids, and endotoxin reactions. Consequently, this disruption could diminish the body’s insulin sensitivity, thereby facilitating the initiation and progression of T2DM (47). However, it is plausible that these alterations in microbial abundance may be a response to environmental stress or high sugar environments aimed at mitigating systemic dysfunction. Conversely, the increased abundance of the *genus Eubacterium oxidoreducens group, genus Oscillospira, genus Ruminococcaceae UCG003, genus Ruminococcaceae UCG010*, and *genus Sellimonas* exhibited a causally negative effect on the risk of T2DM. These findings deepen our comprehension of the intricate interplay between GM and T2DM, potentially paving the way for novel preventive strategies against T2DM. Moreover, it is noteworthy that T2DM itself could influence changes in GM and metabolite profiles.

Based on the findings of this study, there is potential for further investigation into the protective mechanisms of GM in mitigating T2DM. This could lead to the development of foods or beverages enriched with genera such as *Eubacterium oxidoreducens group, Oscillospira, Ruminococcaceae UCG003, Ruminococcaceae UCG010*, and *Sellimonas*, aiming to prevent T2DM. Such endeavors could open up a novel avenue for drug research in the field of T2DM.

The study possesses several strengths. Firstly, it benefits from a large sample size, which minimizes the impact of confounding factors on the results. Secondly, it employs robust methodologies to estimate causal relationships between exposure factors and disease outcomes, thus mitigating the issues of reverse causality often encountered in traditional observational studies. Thirdly, it marks the first identification of the genetic-level association between GM and the risk of T2DM. However, there are certain limitations to consider. Firstly, the outcome data used in the study are derived from European populations, limiting the generalizability of the findings. Future research should aim to validate these results in larger, more diverse populations. Secondly, the available data lack detailed information such as general health status, dietary habits, geographic location, age, and gender, precluding further subgroup analyses. Thirdly, additional mediation MR analyses, particularly focusing on SCFAs, are warranted to validate the findings. Fourthly, considering the multifactorial influence of GM on human health, it is imperative to conduct sequencing verification using clinical samples in future studies. Lastly, the intricate interactions among the gut mycobiome, virome, and microorganisms necessitate further clinical validation to elucidate their specific roles in T2DM. Determining the precise role of these interactions in T2DM pathogenesis represents a crucial research objective.

## 5 Conclusion

There is a causal relationship between GM and T2DM, the *genus Eubacterium oxidoreducens group, genus Oscillospira, genus Ruminococcaceae UCG003, genus Ruminococcaceae UCG010, and genus Sellimonas* are protective factors of T2DM. Metabolites did not appear to act as mediators in the pathway from GM to T2DM.

## Data availability statement

The original contributions presented in the study are included in the article/**Supplementary Material**. Further inquiries can be directed to the corresponding author.

## Author contributions

SS: Conceptualization, Writing – original draft. QZ: Data curation, Project administration, Writing – review & editing. LZ: Methodology, Validation, Writing – review & editing. XZ: Conceptualization, Writing – original draft. JY: Software, Writing – original draft.

## Supplementary material

Supplementary Figure 1 MR analysis between the 418 GM sub-species and T2DM.

Supplementary Figure 2 Funnel plots of sensitivity analysis.

Supplementary Figure 3 Forest plots of LOO sensitivity analysis. The black dot represents the T2DM with increased standard deviation (SD) in GM, which is generated by using each SNP as a separate tool variable. The red dot represents the causal estimation of all SNP combinations by different MR methods. The horizontal line segment represents 95%*CI*. The IVW causal estimate and how the overall estimate (red horizontal line) was disproportionately driven, which is influenced by the removal of a single variant (black horizontal line), were visualized.

Supplementary Table 1 Specific information of IVs used in MR analysis of 11 GM.

Supplementary Table 2 MR estimates of the causal effects of 11 GM on T2DM.

Supplementary Table 3 Sensitivity analysis.

Supplementary Table 4 Relationship between GM related to T2DM and metabolites related to T2DM.
