# Supplementary figures and images for: A two-sample bidirectional Mendelian randomization analysis investigates associations between gut microbiota and type 2 diabetes mellitus
Source: Front Endocrinol (Lausanne). 2024 Mar 1;15:1313651. doi: 10.3389/fendo.2024.1313651 (PMC10940336; doi:10.3389/fendo.2024.1313651)

Supplementary Figure 1

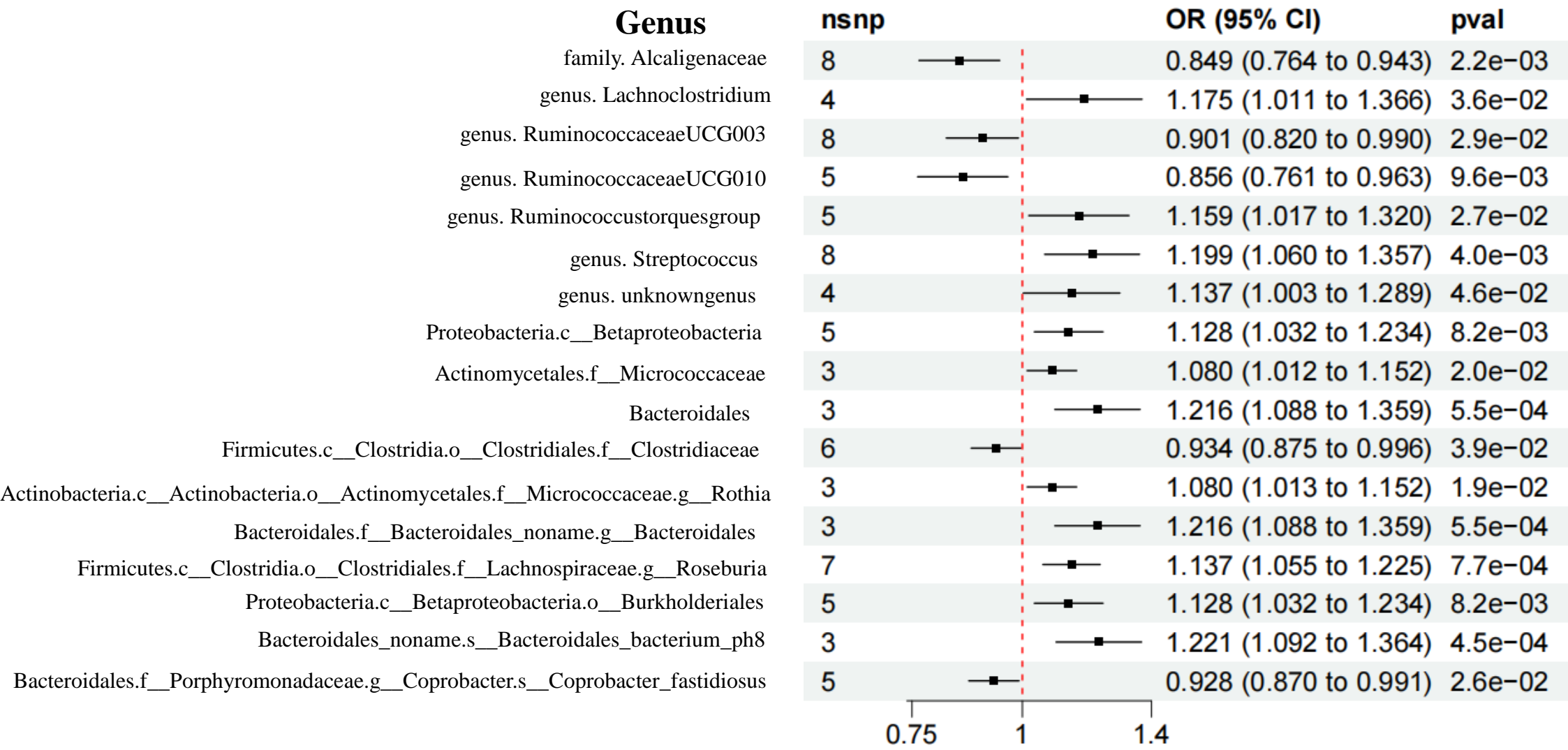

Supplement: Supplementary Figure 1 — MR analysis between the 418 GM sub-species and T2DM. [file Image_1.pdf]
