# Supplementary material for: A two-sample bidirectional Mendelian randomization analysis investigates associations between gut microbiota and type 2 diabetes mellitus
Source: Front Endocrinol (Lausanne). 2024 Mar 1;15:1313651. doi: 10.3389/fendo.2024.1313651 (PMC10940336; doi:10.3389/fendo.2024.1313651)
Supplement: Supplementary Figure 2 — Funnel plots of sensitivity analysis. [file Image_2.pdf]

Supplementary Figure 2

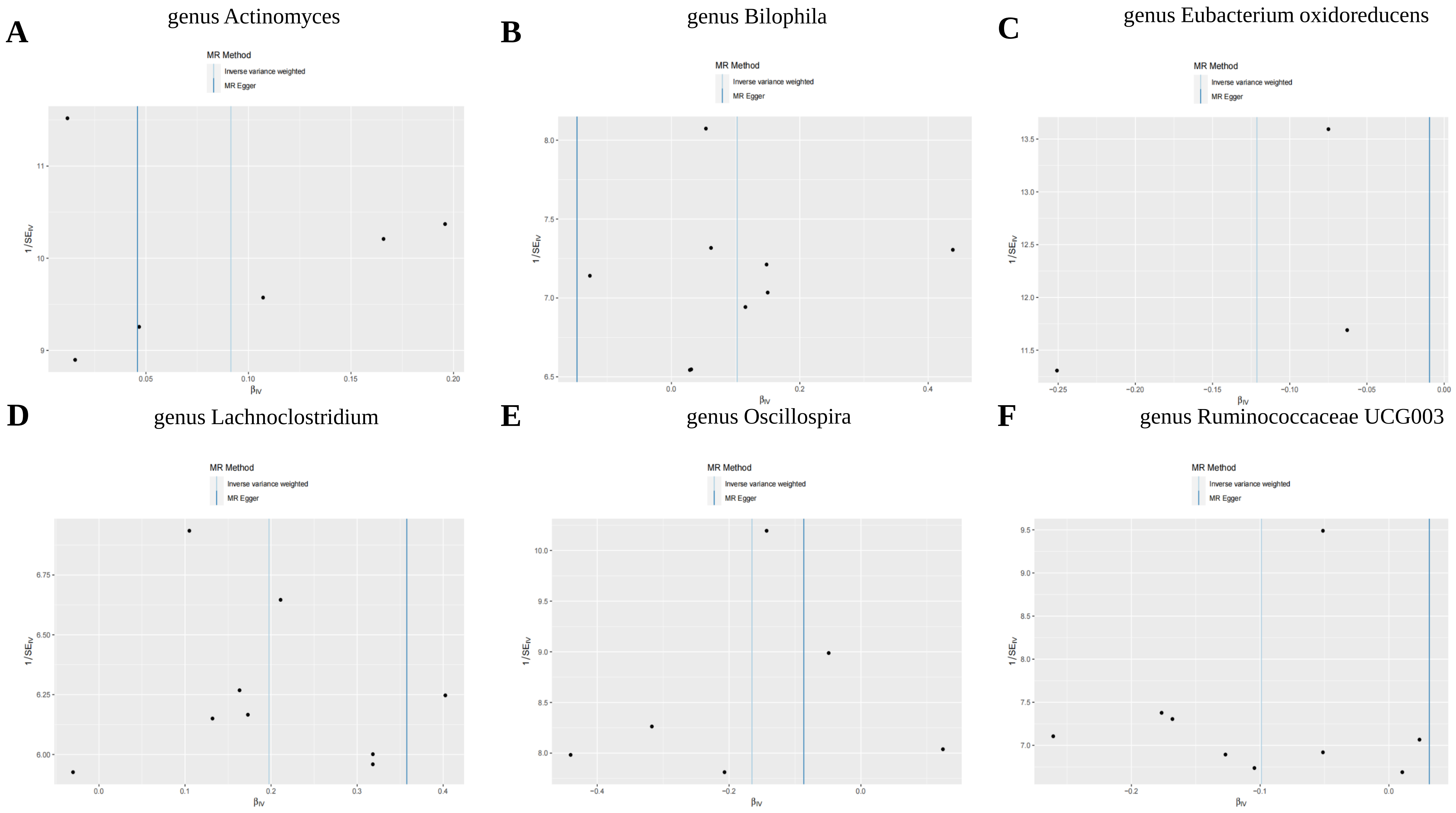

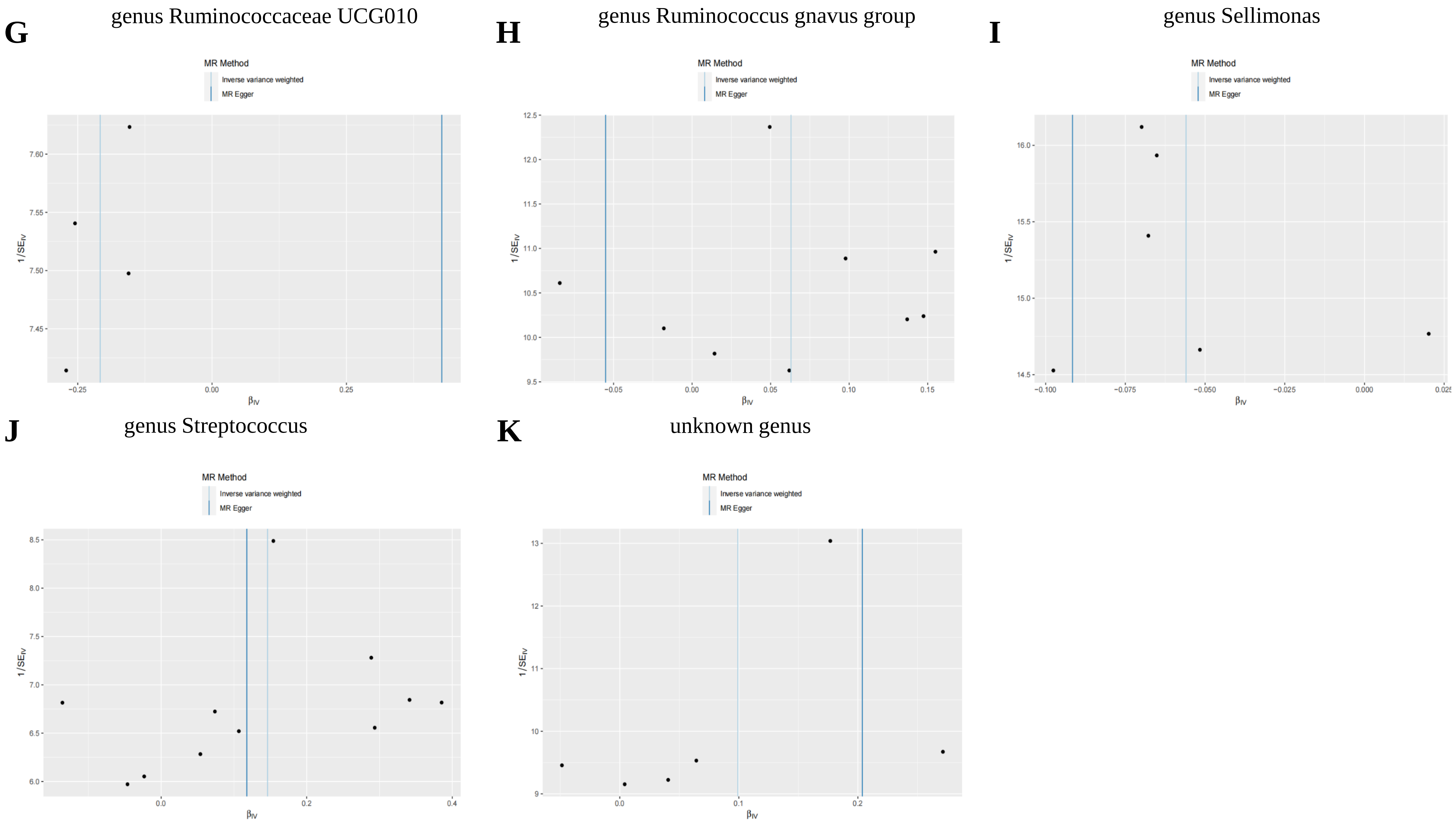

G
genus Ruminococcaceae UCG010
H
genus Ruminococcus gnavus group
I
genus Sellimonas
J
genus Streptococcus
K
unknown genus
MR Method
Inverse variance weighted
MR Egger
1/SE$_{IV}$
β$_{IV}$
