# Supplementary material for: A two-sample bidirectional Mendelian randomization analysis investigates associations between gut microbiota and type 2 diabetes mellitus
Source: Front Endocrinol (Lausanne). 2024 Mar 1;15:1313651. doi: 10.3389/fendo.2024.1313651 (PMC10940336; doi:10.3389/fendo.2024.1313651)

Supplementary Figure 3

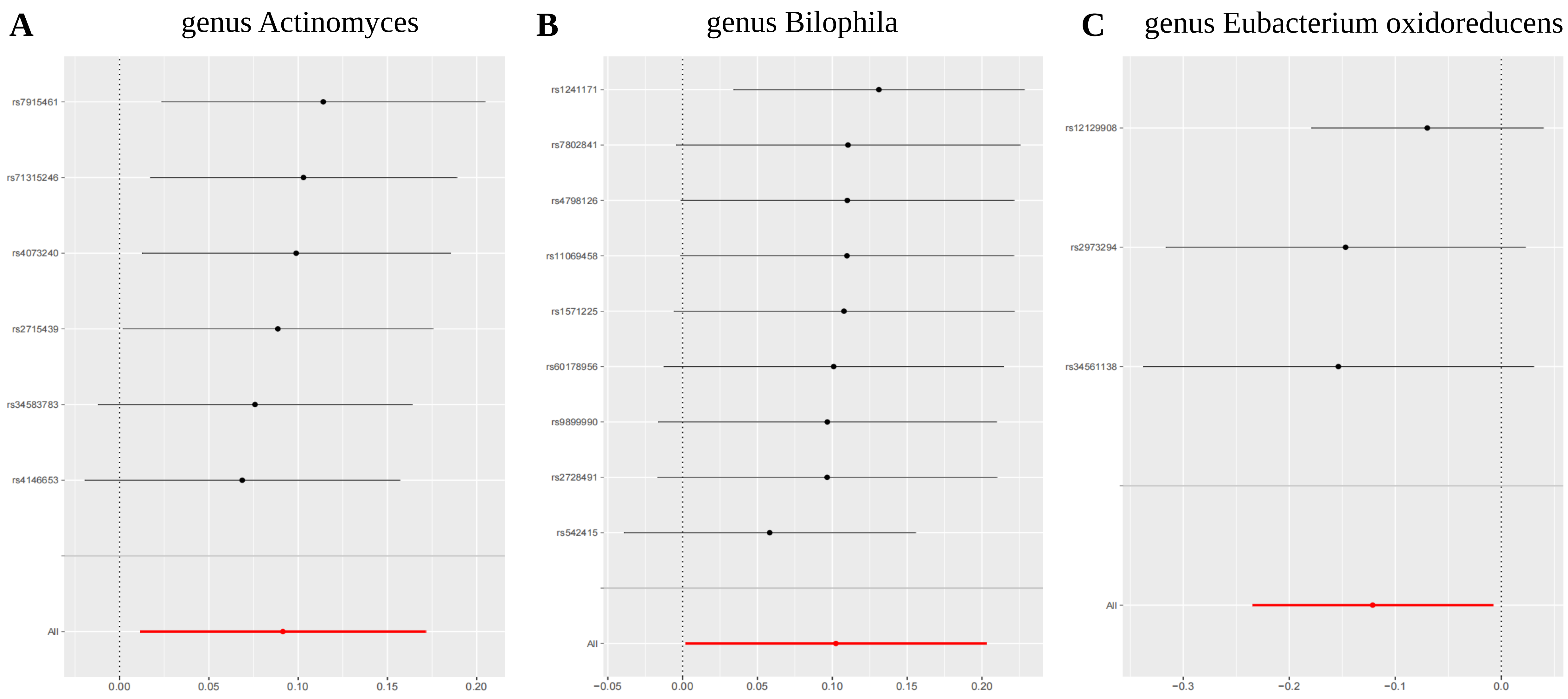

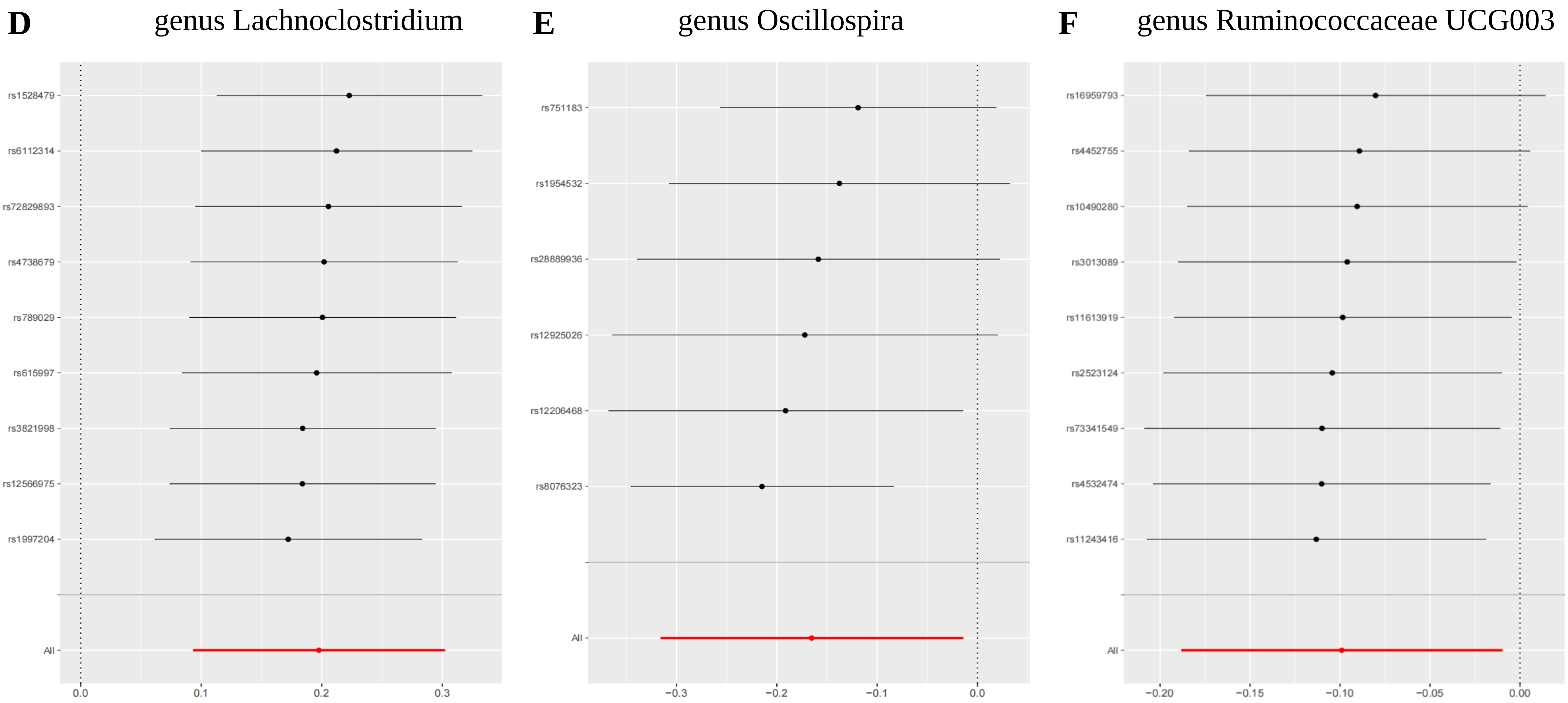
D
genus Lachnoclostridium
rs1528479
rs6112314
rs72829893
rs4738679
rs789029
rs615997
rs3821998
rs12566975
rs1997204
All
0.0
0.1
0.2
0.3
E
genus Oscillospira
rs751183
rs1954532
rs28889936
rs12925026
rs12206468
rs8076323
All
−0.3
−0.2
−0.1
0.0
F
genus Ruminococcaceae UCG003
rs16959793
rs4452755
rs10490280
rs3013089
rs11613919
rs2523124
rs73341549
rs4532474
rs11243416
All
−0.20
−0.15
−0.10
−0.05
0.00

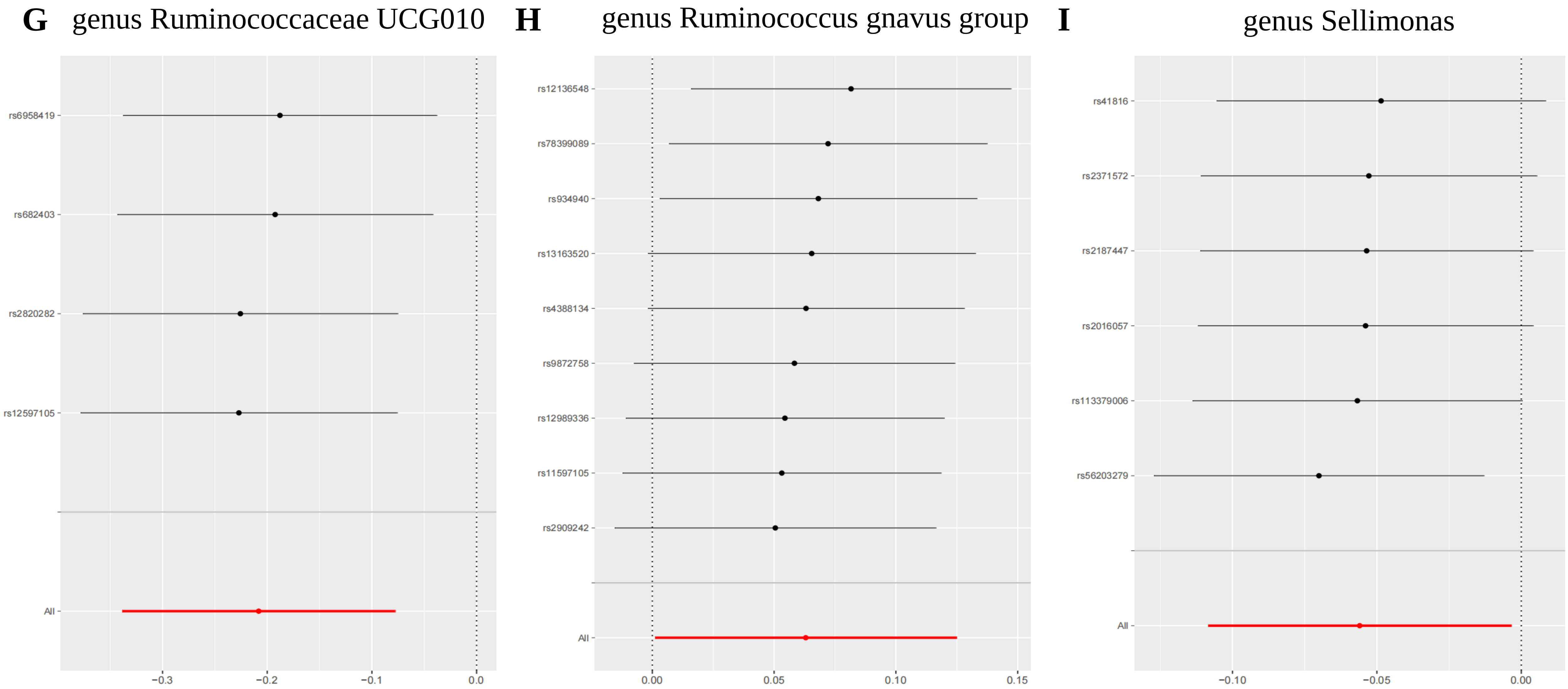
G genus Ruminococcaceae UCG010
rs6958419
rs682403
rs2820282
rs12597105
All
−0.3
−0.2
−0.1
0.0
H genus Ruminococcus gnavus group
rs12136548
rs78399089
rs934940
rs13163520
rs4388134
rs9872758
rs12989336
rs11597105
rs2909242
All
0.00
0.05
0.10
0.15
I genus Sellimonas
rs41816
rs2371572
rs2187447
rs2016057
rs113379006
rs56203279
All
−0.10
−0.05
0.00

J genus Streptococcus

K unknown genus

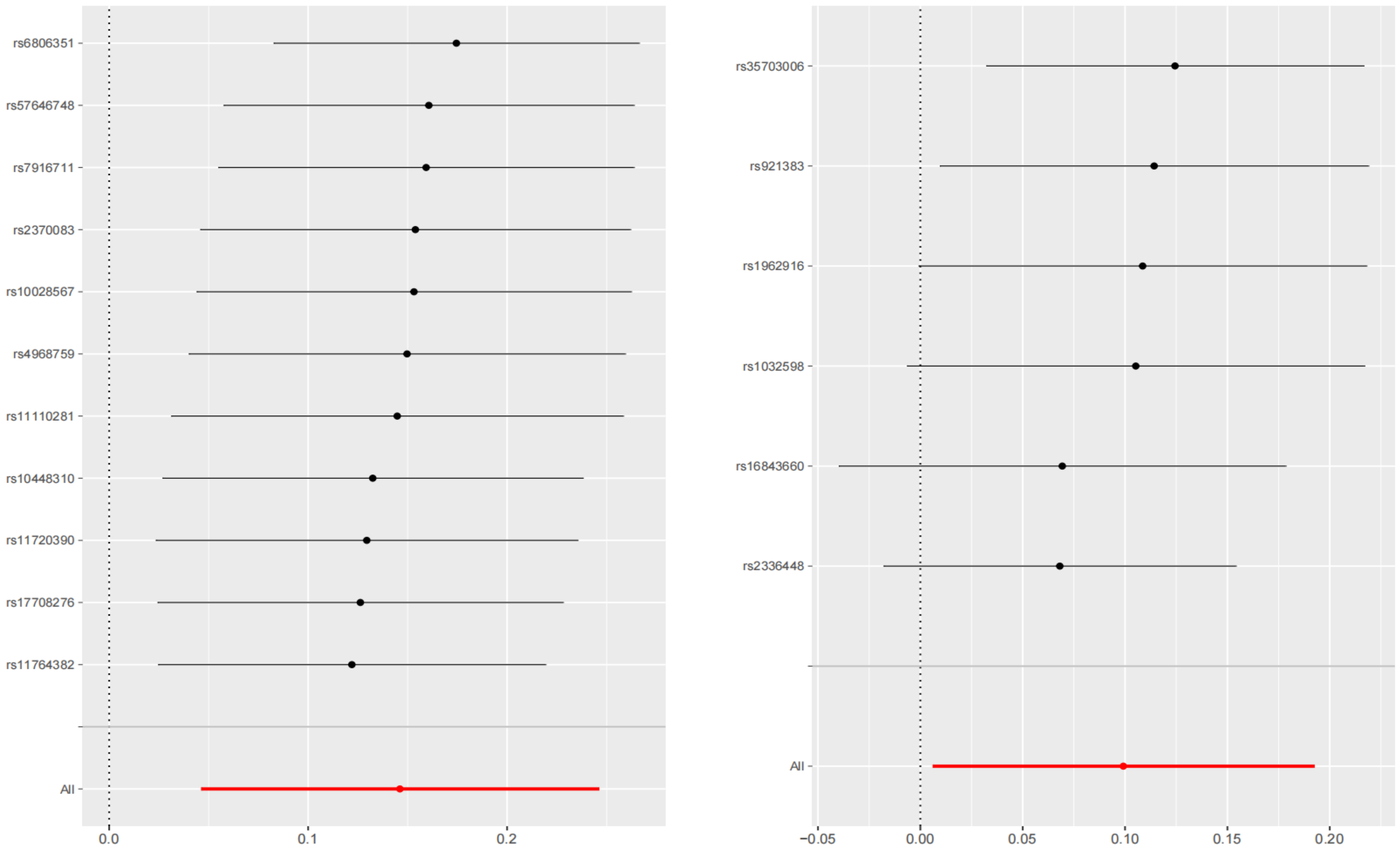

Supplement: Supplementary Figure 3 — Forest plots of LOO sensitivity analysis. The black dot represents the T2DM with increased standard deviation (SD) in GM, which is generated by using each SNP as a separate tool variable. The red dot represents the causal estimation of all SNP combinations by different MR methods. The horizontal line segment represents 95%CI. The IVW causal estimate and how the overall estimate (red horizontal line) was disproportionately driven, which is influenced by the removal of a single variant (black horizontal line), were visualized. [file Image_3.pdf]
